# Supplementary material for: Realization of unpinned two-dimensional dirac states in antimony atomic layers
Source: Nat Commun. 2022 Aug 6;13:4603. doi: 10.1038/s41467-022-32327-8 (PMC9357080; doi:10.1038/s41467-022-32327-8)
Supplement: Supplementary file 1 — Supplementary Information [file 41467_2022_32327_MOESM1_ESM.pdf]

Supplementary Information

Realization of Unpinned Two-Dimensional Dirac States in Antimony Atomic Layers

Qiangsheng Lu, Jacob Cook, Xiaoqian Zhang, Kyle Y. Chen, Matthew Snyder, Duy Tung Nguyen, P. V. Sreenivasa Reddy, Bingchao Qin, Shaoping Zhan, Li-Dong Zhao, Pawel J. Kowalczyk, Simon A. Brown, Tai-Chang Chiang, Shengyuan A. Yang, Tay-Rong Chang, and Guang Bian

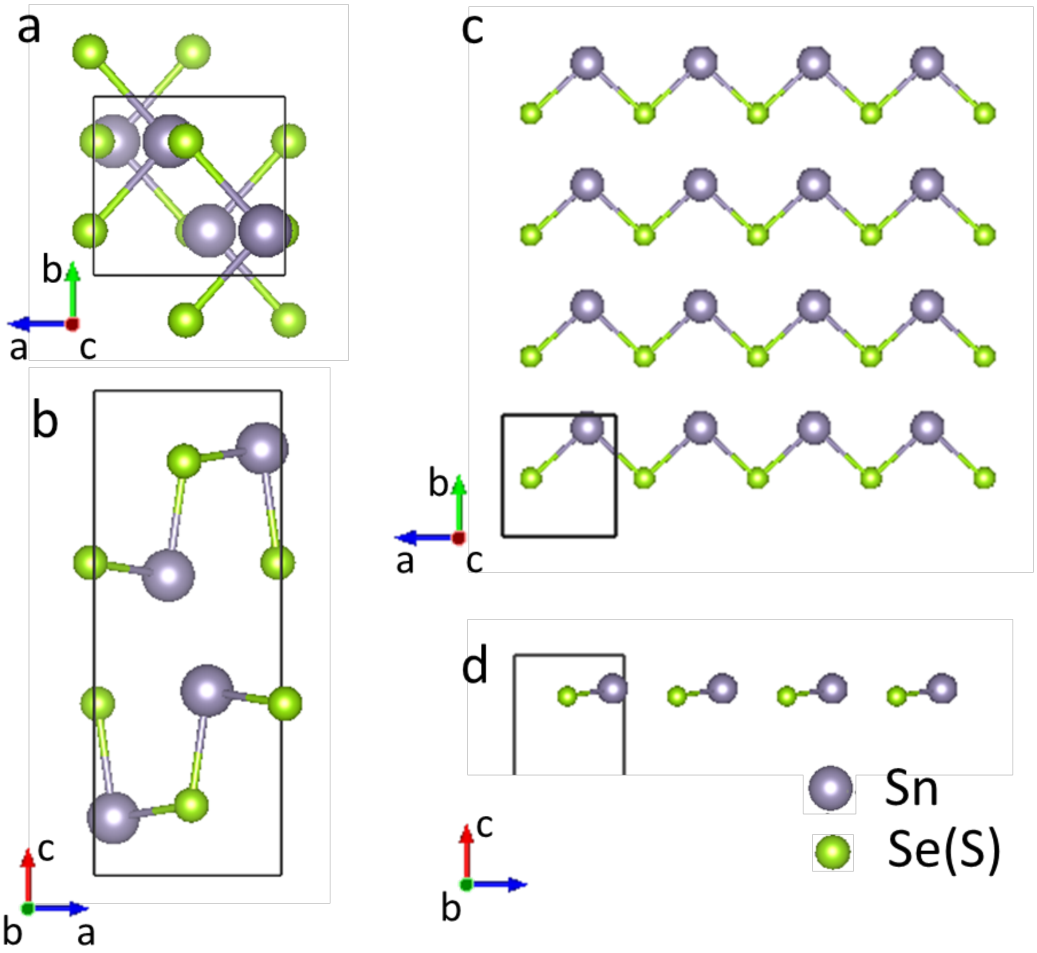

Supplementary Figure1. Lattice structure of SnSe(SnS). (a, b) Top and side view of SnSe(SnS) unit cell. (c, d) Top and side views of SnSe(SnS) surface layer structure.

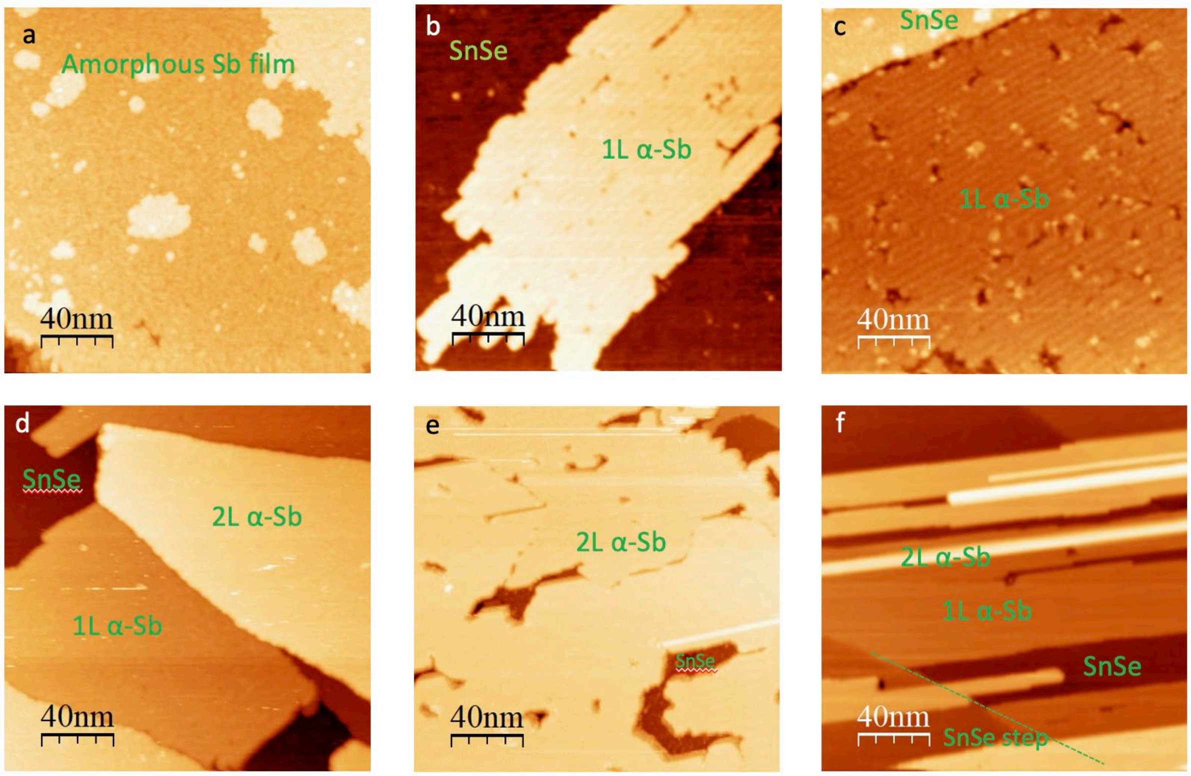

Supplementary Figure 2. STM images of Sb films grown at different temperatures. (a) Amorphous Sb grown in temperature range from 80°C to 110°C. (b-f) α-Sb layers grown at temperatures between 110°C and 120°C. Uniform 1L and 2L α-Sb films can form on SnSe surface. (f) Stripe-shaped islands of α-Sb grown at temperature between 120°C and 140°C.

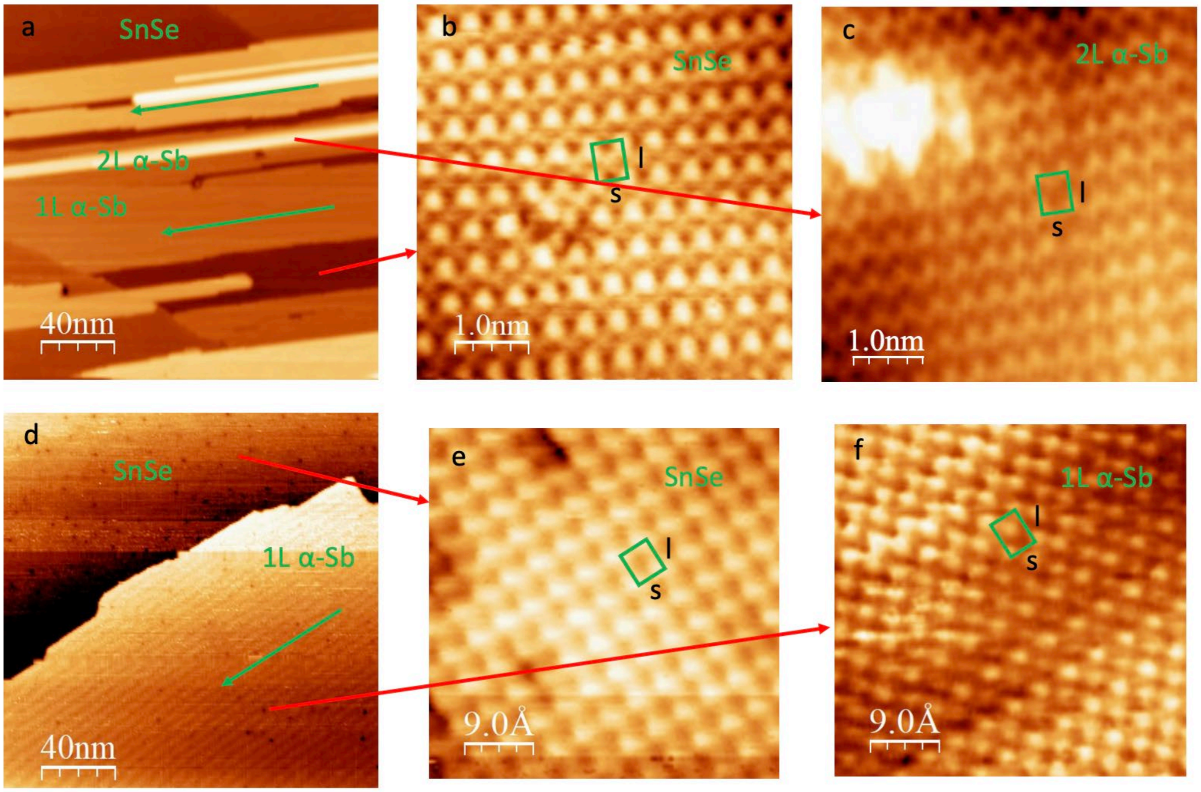

Supplementary Figure 3. STM topography and atom-resolved image of α-Sb films on SnSe
(a, d) STM topography of α-Sb on SnSe. Green arrows in (a) indicate the grown direction of α-Sb stripe-shape islands. The green arrow in (d) shows the direction of Moiré stripes. (b, e) Atom-resolved STM images of SnSe(001) surface. The green rectangular indicate the SnSe unit cell. (c, f) STM images of 2L and 1L α-Sb films. The short edge of α-Sb unit cell is parallel to the short edge of SnSe unit cell. The short edge of α-Sb unit cell is also parallel to the stripe-shaped islands in (a) and Moiré stripes in (d).

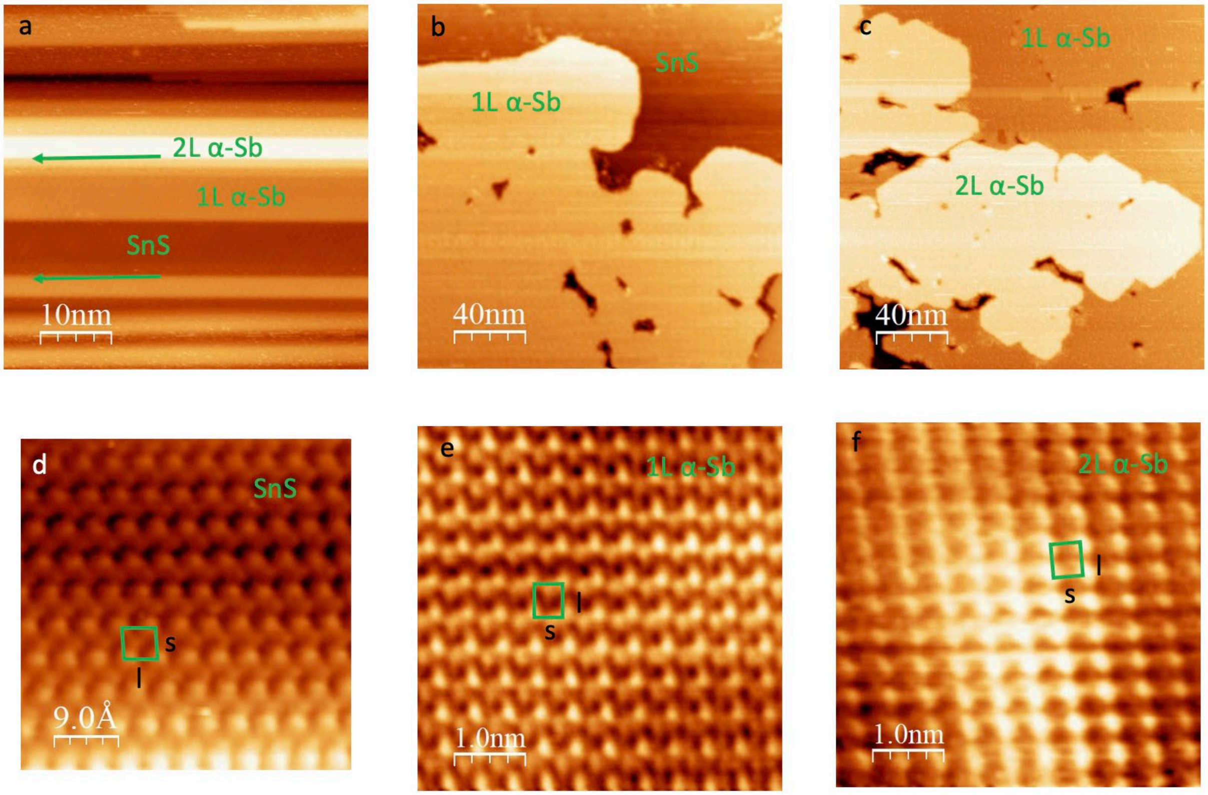

Supplementary Figure 4. STM topography and atom-resolved images of α-Sb grown on SnS

(a, b, c) STM topography of α-Sb on SnS. Green arrows in (a) indicate the growth direction of α-Sb stripe-shaped islands. (d) Atom-resolved STM image of SnS(001) surface. The green rectangle indicates the surface unit cell of SnS. (e, f) High-resolution STM images of 1L and 2L α-Sb on SnS. Green rectangles indicate the unit cell of α-Sb. The short edge of α-Sb unit cell is parallel to the long edge of SnS surface unit cell, which is different from the SnSe case.

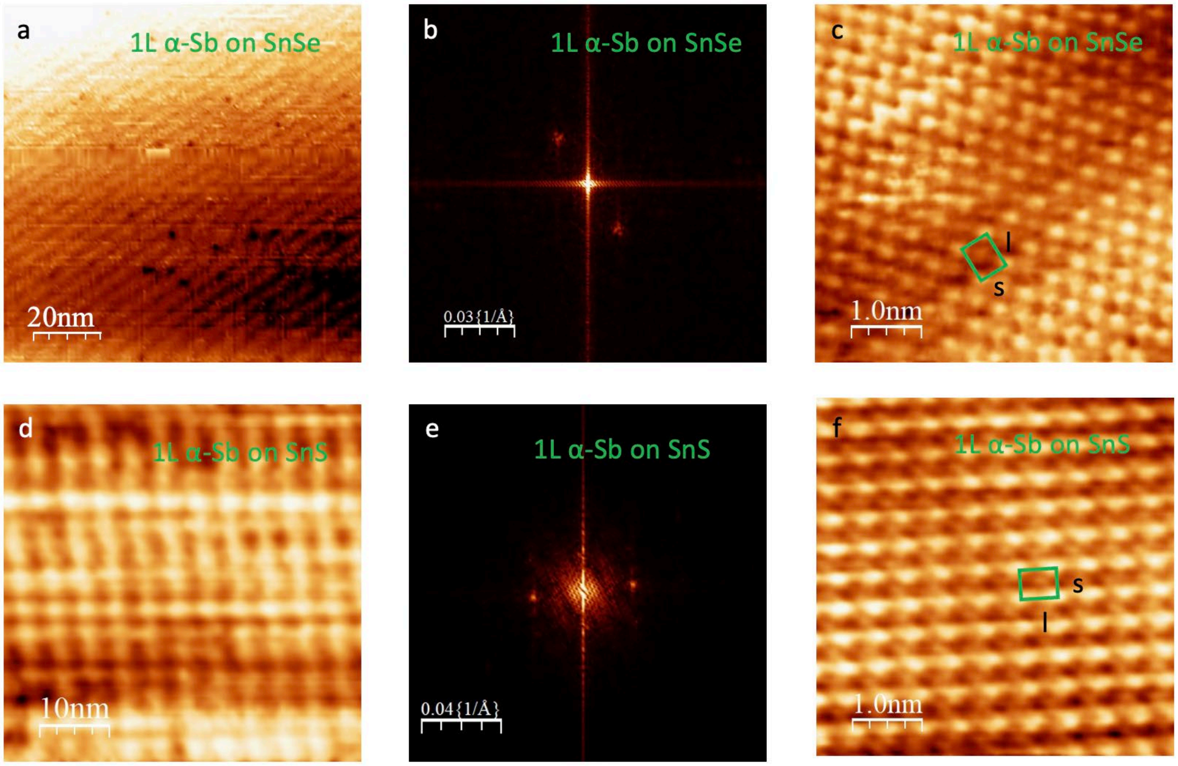

Supplementary Figure 5. Moiré pattern in 1L α-Sb grown on SnSe and SnS (a, d) STM topography of α-Sb on SnSe. Line-shaped Moiré pattern is observed. (b, e) Fast-Fourier transformation (FFT) of (a) and (d). (c, f) Atom-resolved STM images of 1L α-Sb on SnSe and SnS, respectively. The Moiré lines are parallel to the short edge of α-Sb unit cell in both SnSe and SnS samples.

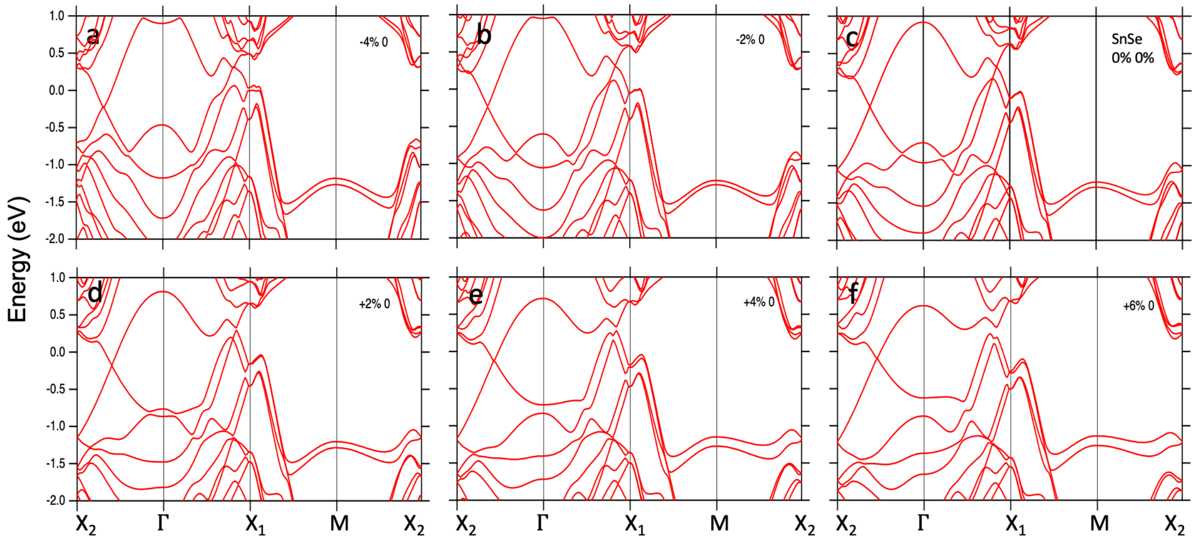

Supplementary Figure 6. Calculated electron band structures of 2L α-Sb with different values of the lattice constant a. (a-f) Band structure with the lattice constant a changed by from -4% to +6%. The reference lattice constants are a= 4.75Å, b=4.15Å, which correspond to the lattice of α-Sb on SnSe measured by STM. Atomic positions are fully relaxed and SOC is included in the band calculation.

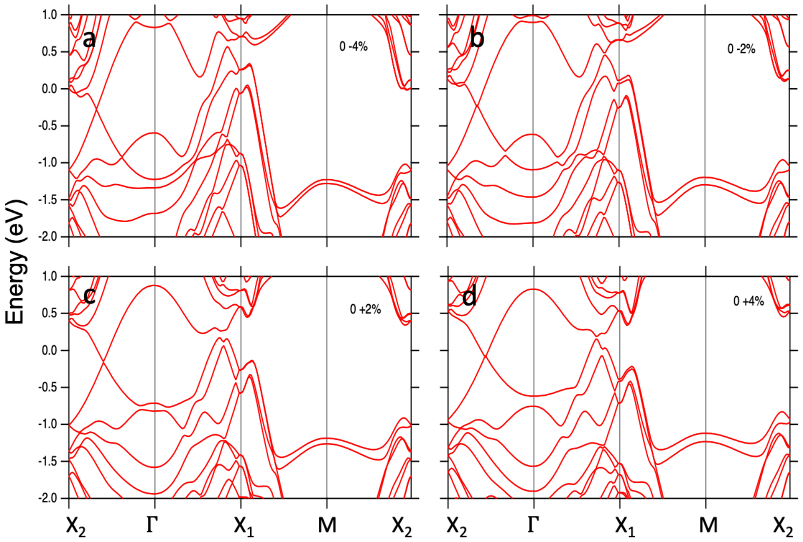

Supplementary Figure 7. Calculated electron band structures of 2L α-Sb with different values of the lattice constant b. (a-f) Band structure with the lattice constant b changed by from -4% to +4%. The reference lattice constants are a= 4.75Å, b=4.15Å, which correspond to the lattice of α-Sb on SnSe measured by STM. Atomic positions are fully relaxed and SOC is included in the band calculation.

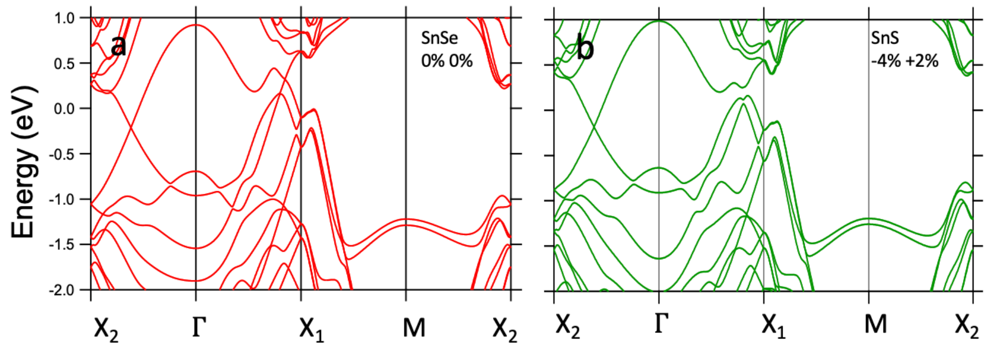

Supplementary Figure 8. Calculated band structure of 2L α-Sb with lattice parameters obtained from SnSe and SnS samples. (a) Band structure of 2L α-Sb with lattice parameters a= 4.75Å, b=4.15Å, which correspond to the lattice of α-Sb on SnSe measured by STM. (b) Band structures of 2L α-Sb with lattice constants a= 4.51Å, b=4.27Å, which correspond to the lattice of α-Sb on SnS.

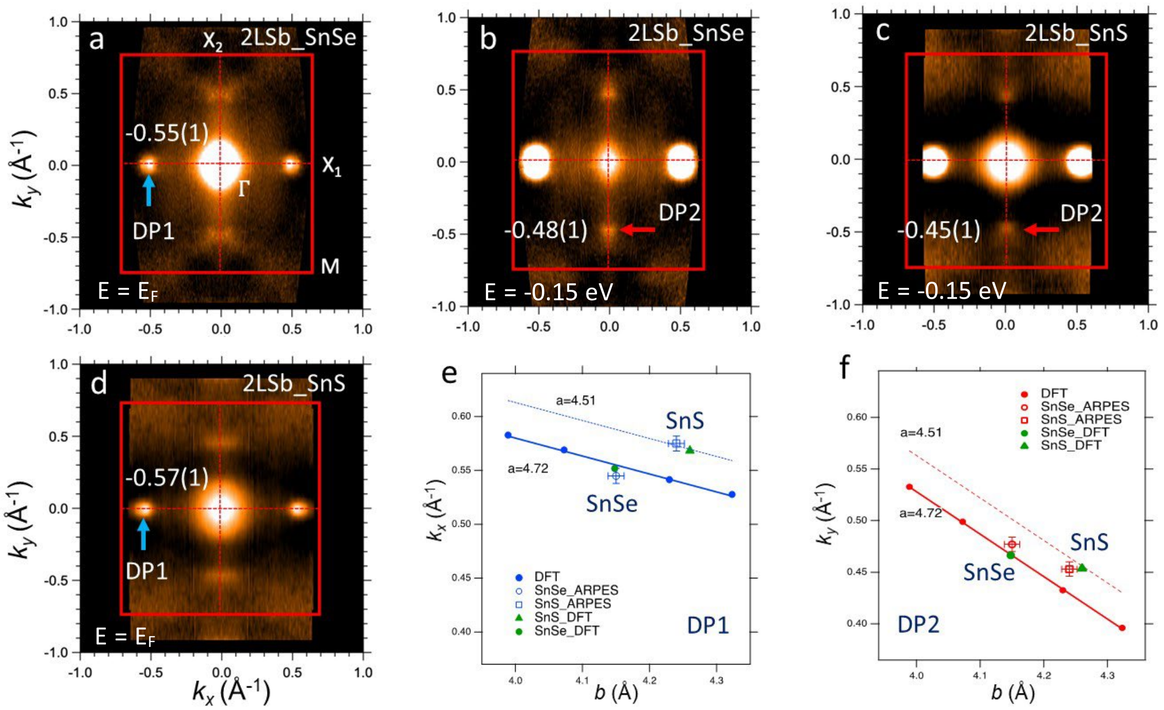

Supplementary Figure 9. (a-d) ARPES iso-energy contour showing the location of Dirac points in 2L α-Sb grown on SnSe and SnS. (e) The calculated location of the Dirac point "DP1" in SnSe and SnS samples. The green solid circles and triangles mark the calculated location of DP1 in SnSe and SnS samples, respectively. The open circles and squares with error bars are the experimental results. (f) Same as (e), but for the Dirac point "DP2" in SnSe and SnS samples.

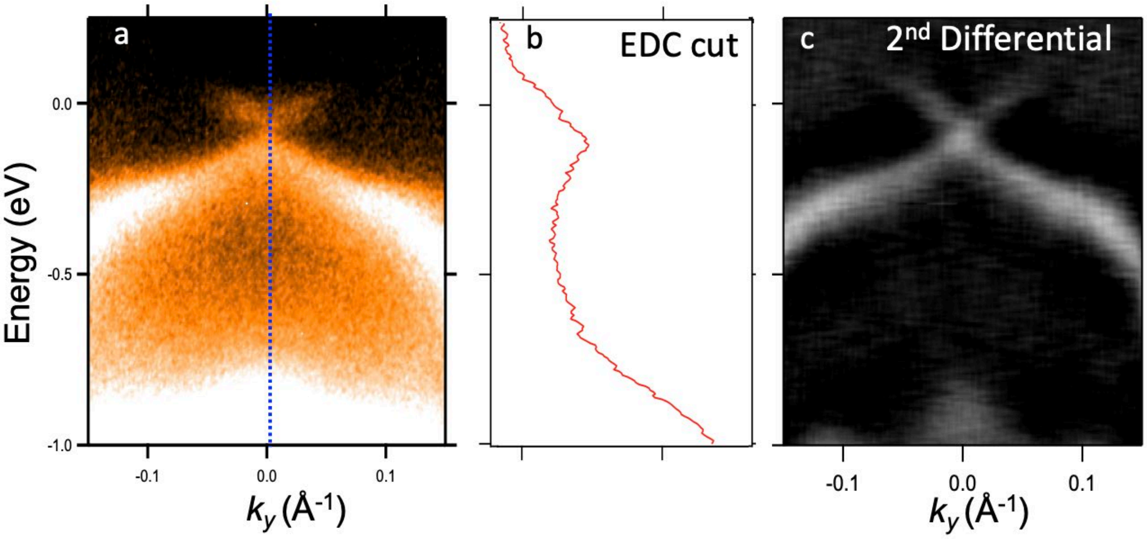

Supplementary Figure 10. (a) ARPES spectrum taken along the line of "cut2" (marked in Fig.5 of the main text), (b) EDC curve taken at the momentum of the Dirac point, and (c) the second derivative of ARPES spectrum in (a). No apparent gap is observed at the Dirac point.

Supplementary Table 1. Lattice constants of SnSe, SnS and α-Sb, and the period of Moiré pattern from the STM results.

	a (Å)	b (Å)
SnSe	4.42	4.10
1L Sb on SnSe	4.81	4.15
2L Sb on SnSe	4.72	4.18
SnS	4.35	3.99
1L Sb on SnS	4.42	4.30
2L Sb on SnS	4.51	4.27

	Moiré FFT ($Å^{-1}$)	Moiré Lattice (Å)
1L Sb on SnSe	0.0194	51.7
1L Sb on SnS	0.0215	45.7
